# Genome-Wide Identification and Expression Analysis of Kinesin Family in Barley (*Hordeum vulgare*)

**DOI:** 10.3390/genes13122376

**Published:** 2022-12-16

**Authors:** Zhaoshun Ye, Zhen Yuan, Huan Xu, Leiwen Pan, Jingsi Chen, Anicet Gatera, Muhammad Uzair, Dawei Xu

**Affiliations:** 1 School of Agronomy, Anhui Agricultural University, Hefei 230036, China; 2 Department of Biochemistry & Cellular and Molecular Biology, University of Tennessee, Knoxville, TN 37996, USA

**Keywords:** barley, kinesin, gene family, hormone response

## Abstract

Kinesin, as a member of the molecular motor protein superfamily, plays an essential function in various plants’ developmental processes. Especially at the early stages of plant growth, including influences on plants’ growth rate, yield, and quality. In this study, we did a genome-wide identification and expression profile analysis of the kinesin family in barley. Forty-two HvKINs were identified and screened from the barley genome, and a generated phylogenetic tree was used to compare the evolutionary relationships between Rice and Arabidopsis. The protein structure prediction, physicochemical properties, and bioinformatics of the HvKINs were also dissected. Our results reveal the important regulatory roles of *HvKIN* genes in barley growth. We found many *cis-* elements related to *GA3* and *ABA* in homeopathic elements of the *HvKIN* gene and verified them by QRT-PCR, indicating their potential role in the barley kinesin family. The current study revealed the biological functions of barley kinesin genes in barley and will aid in further investigating the kinesin in other plant species.

## 1. Introduction

Kinesin superfamily proteins are important players in cellular transport in eukaryotic cells and are involved in complex cytological processes, mainly in protein transportation, mitosis, meiosis, signal transduction, and flagellar motility [1]. Kinesins use microtubules as motor “tracks” to produce energy through ATP hydrolysis, converting chemical energy into bioenergy and providing kinesins with the materials to carry out intracellular activities [2]. Kinesin was first discovered in 1985; kinesin and actin have similar core functions and play a role in processes such as cellular transport and mitosis [3,4]. Today, some new functions of kinesins are being discovered. More and more people are studying kinesins in greater depth.

**Figure 1:**
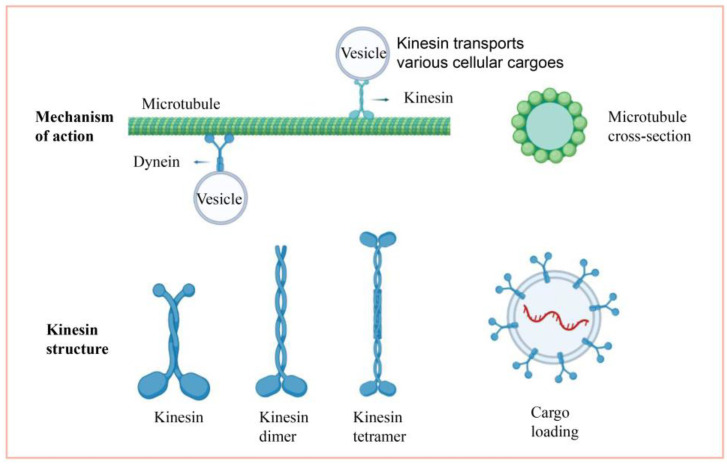
Cartoon description of the kinesin.

Members of the kinesin family have different traits, but the core structure is essentially the same. The kinesin structure consists of two heavy chains (KHC) and two light chains (KLC) [5,6]. The four structural domains of the heavy chain are the motor domain, the dimerization domain, the neck chain, and the tail chain. The cartoon of the kinesin structure is shown in Figure 1; it has two functional sites, the ATP and microtubule binding sites [5,6]. The structural domain is located at the amino terminus of the molecule. This domain is the most conserved of the four structural domains [5,6]. The location of the motor domain in the molecule corresponds to several operating processes as well. N-kinesins are motor structures found at the nitrogen end of the molecule that can move along the positive pole of the vascular towards the cell’s periphery, as opposed to C-kinesins, which move along the negative pole of the microtubule, and M-kinesin, which act to depolymerize the microtubule [1]. The dimerization domain is the molecule’s stem and consists of a coiled-coil shape that plays an important function in controlling the kinesin’s forward motion [7]. The neck chain is a short peptide segment that links the motor and dimerization domains. The tail domain binds the “cargo” and is placed at the carboxy-terminus of the kinesin. This area is not extremely conserved, resulting in the variety of the “cargo” it carries. Synaptic vesicle membranes, mitochondria, axons, mRNAs, and lysosomes, for example, are all carried by the tail [8,9]. Furthermore, two light chains are attached to the “cargo” membrane organelles in the tail and migrate along the microtubules. According to these findings, the high conservation of the motor domain and the low conservation of the tail reflect the various activities of proteins in the same kinesin family.

At present, kinesins have been classified into 14 groups (kinesin1~kinesin14) based on their conserved sequences [1]. Kinesin-1 to Kinesin-3 proteins, when not transporting cargo, reduce ATP consumption and microtubule utilization through an autoinhibitory conformation [10]. Kinesin-1 and kinesin-2 mainly form dimers for autoinhibition, whereas kinesin-3 completes autoinhibition by interlocking the neck and motor structures [11]. AtKRP125b belongs to the kinesin-5 family and is mainly involved in mitotic processes in Arabidopsis [12]. Similarly, knockout of the *ATK1* gene will lead to abnormal mitosis and failure to form normal microspores in Arabidopsis [13]. Kinesin-12E regulates mid-mitotic spindle function and plays an important role in mitosis in Arabidopsis [14]. Changes in the structural domain of kinesin-2 can lead to abnormal pollen wall development in Arabidopsis [15]. In addition, kinesin is also involved in different processes, such as drought stress [2,16], nuclear division [17,18], and vesicle formation in the root periphery [19]. In rice, Kinesin-5 is the primary kinetic motor of the mitotic spindle [20]. Kinesin-14 can enter the nucleus in response to cold [21], and Kinesin-4 can regulate granule width by controlling cell proliferation [22]. *SRS3*, a Kinesin-13 protein subfamily gene, was discovered to be capable of regulating seed length. *SAR1*, on the other hand, is a kinesin gene that, like M-kinesin, can depolymerize cellular microtubules to affect seed shape and size [23]. However, the kinesin family’s role in barley has received little attention. As a result, the role of kinesins in barley growth and development merits further investigation.

Barley is the fourth largest crop in the world, followed by rice, maize, and wheat. Barley is grown on a large scale worldwide because of its high resistance and adaptability. Barley is highly utilized in many aspects of life, such as fodder, medicine, and brewing. Barley quality and yield are susceptible to key factors in the growth and development process. Among these are seed development, organ development, and cell division, all of which are influenced by the kinesins [22,24,25]. There are currently no specific reports on the study of barley kinesin gene families and their functions during various developmental stages. To predict the role of barley kinesin families in growth and development, we compared the evolutionary features of kinesin families in plants such as barley, rice, and Arabidopsis. The biological functions, expression patterns, and protein structures of the forty-two barley kinesins screened were also studied. The roles of barley *kinesin* genes in barley growth were further revealed, and some theoretical guidance for future studies was provided by analyzing the evolutionary genetic relationships, including gene structures, chromosomal location, conserved motifs, and *cis*-regulatory elements analysis.

## 2. Materials and Methods

### 2.1. Materials and Data Sources

Golden Promise barley was planted in the experimental field of the campus (31.85° N, 117.26° E) at a temperature of 15–25 °C. The plant genomic database phytozome (phytozome-next.jgi.doe.gov/ accessed on 9 October 2021) was used to retrieve the AtKINs, OsKINs, and HvKINs protein sequences [26]. Using PF00225 (http://pfam.xfam.org/ accessed on 9 October 2021) as the kinesin signature domain to perform the Hidden Markov Model (HMM) algorithm [27,28], set a threshold of E-value < 10^−10^ and screened for the forty-two *HvKINs* as candidates.

### 2.2. Analysis of Barley HvKIN Family Proteins

The primary physicochemical properties, such as molecular weight, theoretical isoelectric point, charge residue number, total atomic number, instability coefficient, lipolysis index, and hydrophobicity index, were evaluated using the ProtParam tool (web.expasy.org/compute_pi/ accessed on 16 October 2021). Protein subcellular localization is predicted using TargetP (https://services.healthtech.dtu.dk/service.php?TargetP-2.0 accessed on 16 October 2021) and WoLF PSORT (http://www.genscript.com/wolf-psort.html accessed on 16 October 2021) [29]. The 3D protein structure of the HvKINs was predicted by the PHYRE2 protein fold recognition server (www.sbg.bio.ic.ac.uk/phyre2/html/page.cgi?id=index accessed on 16 October 2021).

### 2.3. Phylogenetic Analysis of Barley HvKIN Proteins

The multiple sequence alignments were performed by MEGA-X software (www.megasoftware.net/ accessed on 23 October 2021). The Clustal W tool in Multiple sequence comparisons of protein sequences was performed using ClustalW2 with parameters set to Gap Opening Penalty = 10, Gap Extension Penalty = 0.2, and Delay Divergent Cutoff = 30%. The evolutionary tree was then constructed using the maximum likelihood method with parameters set to Bootstrap = 1000 [30,31].

### 2.4. Chromosomal Localization, Gene Structure, Conserved Structural Domains, Promoter Analysis, and Covariance Analysis of the HvKIN Gene

The Barley Genome Database (phytozome.jgi.doe.gov/pz/portal.html accessed on 17 December 2021) was used to download the CDS and protein sequences. Using the barley IPK website (apex.ipk-gatersleben.de/apex/f?p=284:10 accessed on 17 December 2021) to predict the positions of each gene. Gene Structural analysis and chromosomal localization of the *HvKIN* genes were completed using TBtools software (bio.tools/tbtools accessed on 20 December 2021) [32]. The conserved functional, structural domains in the *HvKIN* protein sequences were completed using MEME (meme-suite.org/meme/tools/meme accessed on 20 December 2021). Two kb upstream of each gene genomic DNA sequence were retrieved from the barley genome, and the *cis*-regulatory elements were analyzed by the PlantCARE online tool [33,34]. Ka/Ks were predicted using the website tool (bio.tools/kaks_calculator accessed on 12 January 2022).

### 2.5. Expression Modelling Analysis of the HvKINs, QRT-PCR Analysis, and Hormone (ABA and GA3) Treatment

To investigate particular expression profiles of *HvKINs* in different barley tissues and phases of development, the results of RNA-Seq from various development stages were acquired from the IPK website, and heatmaps were plotted by the online TBtools tool. The expression level of the forty-two genes was verified by QRT-PCR in root seedlings (2 cm stem stage), stems (30 days), leaves (30 days), immature fruit (2-week-old sowings), and mature seeds of barley, using TRNzol (Invitrogen, Waltham, MA, USA) reagent to extract the total RNA. RNA quality and quantity parameters of all the samples were verified by Nano Drop 1000 spectrophotometer (Thermo Fisher Scientific, Waltham, MA, USA). DNA contamination was removed by reverse transcription using DNase I (TaKaRa), and first-strand cDNA was generated from one microgram of total RNA using HiScript III Reverse Transcriptase R302 (Nanjing Vazyme Biotech Co., Ltd., Nanjing, China). QRT-PCR procedures and data analysis were performed as previously described [35,36]. HvACTIN (HORVU1Hr1G074350.1) was chosen as an internal reference gene [37]. The primers used for QRT-PCR are listed in Supplementary Table S1.

Barley plants growing at 30 days were treated with 100 uM ABA [38] and 50 mg/L GA3 [35], while the control was sprayed with an equal amount of pure water. Leaves were selected at 0 h and 12 h, and the RNA was extracted and estimated by QRT-PCR to detect *HvKIN* gene expression.

### 2.6. Subcellular Localization of the HvKINs in Barley

To investigate the transient expression of *HvKINs* in tobacco leaves, full-length *HvKIN* CDS was PCR amplified using the primers containing the Bgl II and Spe I restriction enzymes (sequences in Supplementary Table S2) and ligated into the vector pCAMBIA1301-eGFP digested with Bgl II and Spe I to generate *1301-35Spro::HvKINCDS-GFP.* The *1301-35Spro::HvKINCDS-GFP* fusion protein translation initiates at the start codon in the Nco I restriction enzyme site, which is located before Bgl II and causes no frameshift change of the HvKIN proteins. Constructed vectors were transformed into *Agrobacterium tumefaciens* GV3101 and infiltrated into leaves of four-week-old tobacco plants, and after 48 h dark incubation, the fluorescent signal was observed using a confocal microscope (Leica TCS SP5) in the tobacco leaf epidermis. The detailed observation by microscope was performed as previously described [39].

## 3. Results

### 3.1. HvKIN Genes Identification in the Barley

To fully understand the evolutionary history of the KIN family of barley, BLAST analysis and conserved structural domain analysis were performed using kinesin sequences from *Oryza sativa* and *Arabidopsis thaliana*. HMM searches were validated through the Pfam website and forty-two *kinesin* genes were recognized from the barley Morex genome in total and designated HvKIN1-HvKIN42 (Supplementary Table S3). In addition, we have analyzed the physical properties of each kinesin by bioinformatics prediction (Supplementary Table S3). Briefly, the HvKIN protein length ranged between 210 to 3016 amino acids, and the protein molecular weight (MW) ranged from 23.71 kDa~341.86 kDa. The isoelectric point (pI) is predicted to range from 4.85~9.82, with 71% (30/42) having an acidic isoelectric point of less than 7. Because the hydrophobicity indices of the family proteins are all negative, this indicates that the HvKINs are all hydrophilic proteins. It follows that all forty-two HvKINs proteins are stable.

**Figure 2:**
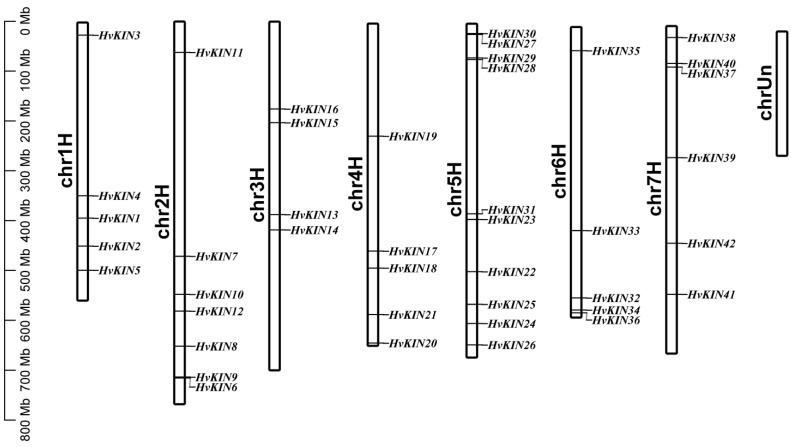
*KIN* genes distribution on barley chromosomes. Note: Each bar has a chromosomal number above it. The base pair scale is on the left (bp).

As we can see from the results of gene distribution, *HvKIN* genes are localized on seven chromosomes and show an uneven distribution. Among them, 10 of these *HvKIN* genes are located on chromosome five, chromosome three is the least distributed, with only four genes, and the other chromosomes have 5–7 kinesin genes distributed on them (Figure 2).

### 3.2. Phylogenetic Analysis of HvKINs Proteins

**Figure 3:**
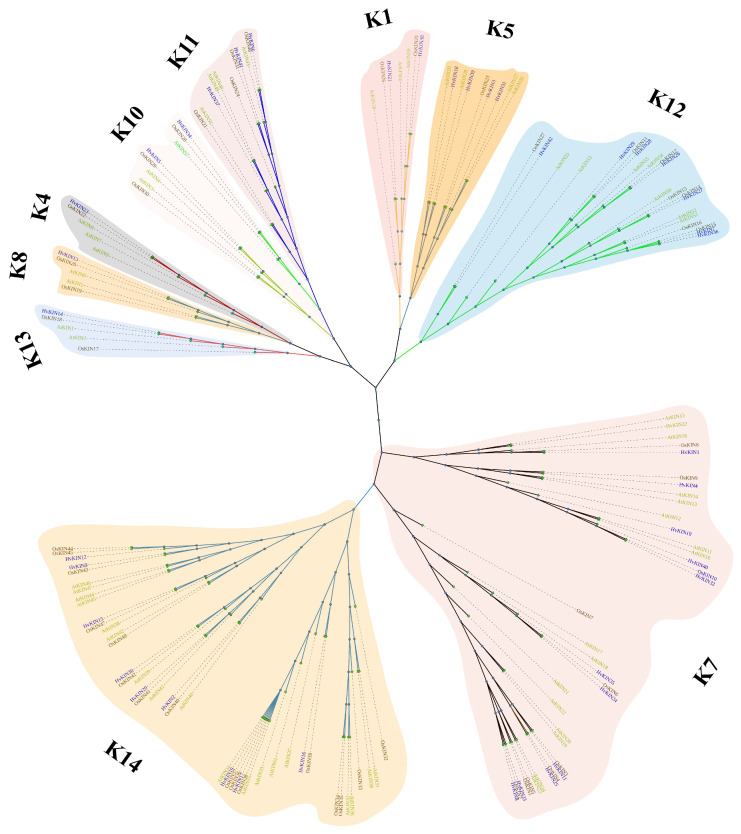
Phylogenetic analyses of KIN protein. Using MEGA7.0 software (Temple University, Philadelphia, Pennsylvania, USA.) to generate the KINs’ phylogenetic tree with the default settings. K1, K4, K5, K7, K8, K10, K11, K12, K13 and, K14 are 10 subfamilies of kinesins. Different colors were used to identify the 10 groupings. *At*, *A. thaliana*; *Os*, *O. sativa*; *Hv*, *H. vulgare*.

To investigate the evolutionary history and functional associations of kinesin family proteins, a maximum likelihood phylogenetic tree was constructed by MEGA X (https://www.megasoftware.net/ accessed on 9 October 2021), using the full-length amino acid sequence of KINs from 42 members in barley, 48 members in rice and 61 members in Arabidopsis (Figure 3).

The phylogenetic tree shows that the kinesins of the three different species can be separated into ten subgroups, namely subgroups K1, K4, K5, K7, K8, K10, K11, K12, K13 and, K14 (Figure 3). Of these, subgroup K14 belongs to the largest subfamily, consisting of 43 kinesins. Subgroup K7 is the second largest family with 37 kinesins. Subgroups K4 and K13 are the smallest subfamilies, consisting of only 5 kinesins. In the largest subfamily, K14, there are 9 barley kinesins, and in the smallest subfamily, K4 and K13 there is 1 barley kinesin. Phylogenetic tree analysis also showed that barley, rice, and Arabidopsis were broadly consistent in kinesin evolution. There was no significant variability between monocotyledons and dicots, fully reflecting plants’ conserved nature of kinesins.

### 3.3. Analysis of Gene Structure and Conserved Motif Distribution of Barley HvKINs

**Figure 4:**
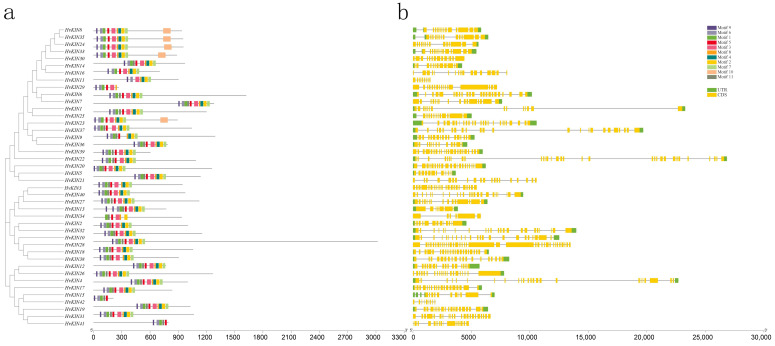
HvKINs structure and conserved motif distribution analysis. (**a**). The barley *HvKIN* proteins’ motif composition. The motifs, which are shown in variously colored boxes, were identified by MEME 5.0.1 in the *HvKIN* proteins. (**b**) The yellow and green bars represent the CDS and the untranslated region (UTR), respectively, while the black line represents the intron.

To investigate the structural features of *KINs* in barley, the conserved motifs were constructed by MEME software. The result shows there have 11 similar higher conserved motifs (Figure 4a, Supplementary Figure S1, Supplementary Table S4). Some barley kinesins possess the conserved sequences Motif1 (FAYGQTGSGKT) for the ATP-binding site, Motif2 (HVPYR), Motif3 (SSRSH) and Motif8 (VDLAGSE) for the microtubule-binding site [40,41,42]. Using SMART analysis of conserved motifs revealed that Motif1, Motif2, Motif3 and Motif8 are KIS superfamily structural domains, normally microtubule-dependent molecular motors that play an essential role in intracellular transport of organelles and cell division events [43].

To understand the structural features of barley kinesin genes, we analyzed the gene structure and intron/exon arrangement of *HvKINs* by downloading barley whole genome annotation GFF3/GTF files and using TBtools software (Figure 4b, Supplementary Figure S1). The results showed that the number of introns in all genes ranged from 7 to 38, with *HvKIN28* containing 38 introns and *HvKIN11* only 7 introns. Clearly, the gene structures differed considerably between the kinesin families.

### 3.4. Analysis of Cis-Acting Elements of the HvKINs Gene

**Figure 5:**
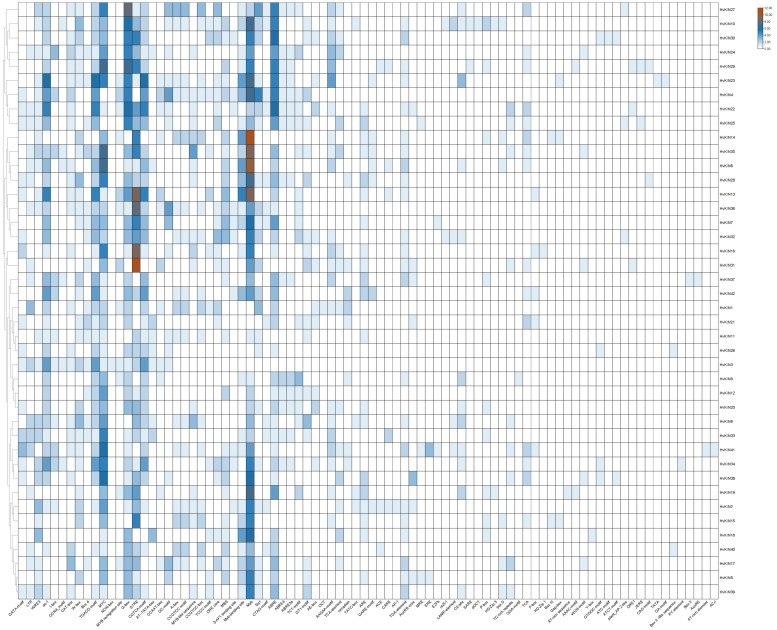
Cis-acting element structures in promoter regions of barley *HvKIN* family members.

To understand the evolutionary features of the *HvKINs* gene family, we performed a predictive analysis of the sequence 2000 bp upstream of the *HvKINs* gene transcription start site (Figure 5, Supplementary Figure S2). The *HvKINs* family contains 3049 cis-acting elements, with a variety of cis-acting elements a high degree of variation exists between genes. Two essential elements, CAAT-BOX and TATA-BOX, commonly found in eukaryotes, were removed and the rest of the elements were plotted (Figure 5). Among all the cis-acting elements, a large number of hormone-response-related regulatory elements are contained, mainly hormone (TCA-element and GARE-motif), gibberellin (TATC-box), salicylic acid (TCA-element), abscisic acid (ABRE), methyl jasmonate (CGTCA-motif and TGACG-motif) maize alcohol-soluble protein (O2-site), etc. There are also certain abiotic stress-responsive elements, including anaerobic (ARE), photo-responsive (ATC-motif, TCT-motif, G-box, GT1-motif, Sp1, MRE and Box 4), low temperature (LTR), and drought (MBS) (Figure 5). The expression of *HvKINs* genes is influenced by various signals, suggesting that kinesin plays an important function in normal plant growth and development and maintains the balance of hormone metabolism in vivo.

### 3.5. Evolutionary Analysis of the HvKIN Genes

The duplication events may illuminate the mechanism of the expansion of the *HvKIN* gene family. Gene families formed by tandem duplication are mainly located on the same chromosome and are similar in sequence and function; genes formed by duplication of chromosomal segments are more distant and are usually on different chromosomes. Calculation of non-synonymous substitutions (Ka) to synonymous substitutions (Ks) rates were used to infer the size of the selection constraint and determine whether a protein-coding gene is subject to the selection pressure of gene duplication gene pairs in the evolution process. If Ka/Ks > 1, a positive selection effect is considered to exist; if Ka/Ks = 1, a neutral selection effect is assumed to exist; if Ka/Ks < 1, a negative selection effect (Purification effects or purification options) [44].

**Figure 6:**
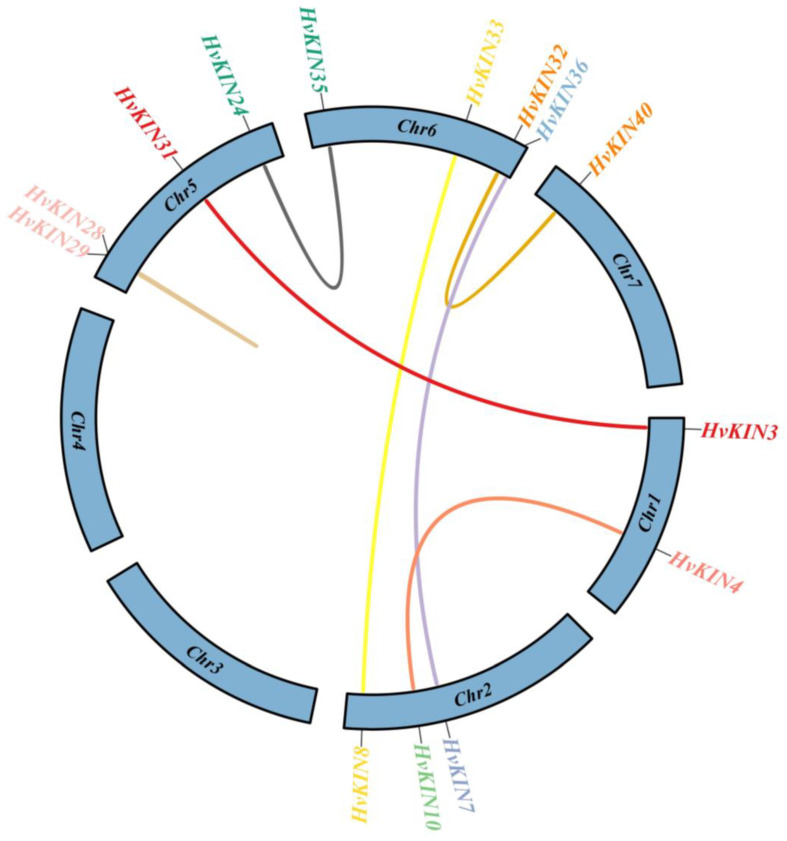
The members of the barley *HvKIN*, along with their corresponding gene pairs and chromosomal locations. Different colored lines represent the two genes’ connection in terms of replication. The name of the linked gene and its location on the chromosome are listed in the outer circle. TBtools v1.082 (South China Agricultural University, Guangzhou, China) was used to create this image.

To determine the selection influence on the evolution of the *HvKINs*, seven pairs of homologous genes were screened in a study of the *HvKINs* gene family in barley (Figure 6, Supplementary Table S5). Our results show the Ka/Ks values were all < 1, which means *HvKINs* genes were primarily determined by stabilizing selection.

### 3.6. Structural Analysis of the 3D Protein of the HvKINs

**Figure 7:**
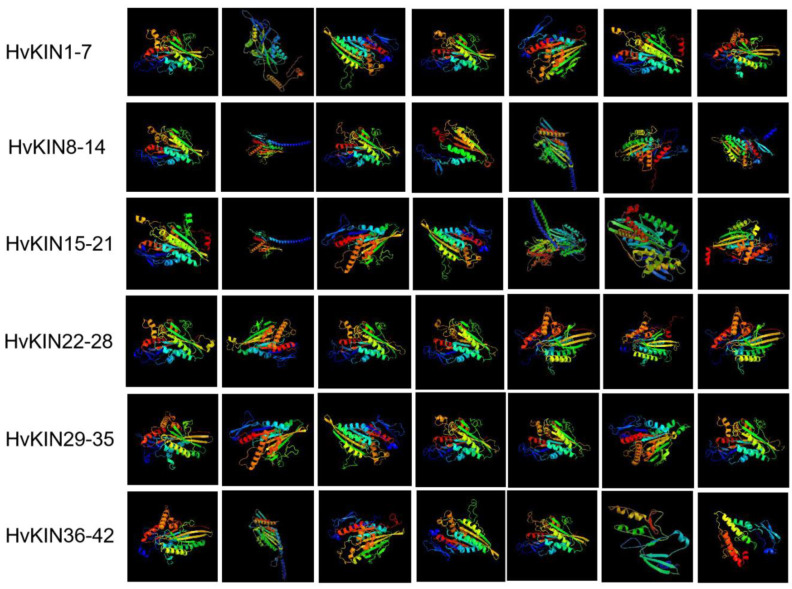
Structure of the HvKINs gene 3D protein.

According to the protein 3D structure prediction website (phyre2, http://www.sbg.bio.ic.ac.uk/~phyre2/html/page.cgi?id=index accessed on 9 March 2022), forty-two HvKIN proteins have a complex protein secondary structure and are mainly composed of α helix and Random coil (Figure 7). The percentage of α helix in the family ranges from 27.14% (*HvKIN42*) to 73.09% (*HvKIN22*), and the percentage of Random coil ranges from 17.31% (*HvKIN28*) to 52.56% *(HvKIN14*) (Figure 7). In addition, β-turn (β turn) and extended strand (Extended strand) are included, with β-turn ranging from 1.96% *(HvKIN27*) to 7.62% (*HvKIN42*) and extended strand ranging from 5.97% (*HvKIN22*) to 26.67% (*HvKIN42*) of the members. This indicates that the family proteins are mainly composed of α-helices and irregular coils.

### 3.7. Tissue-Specific Expression of HvKINs

**Figure 8:**
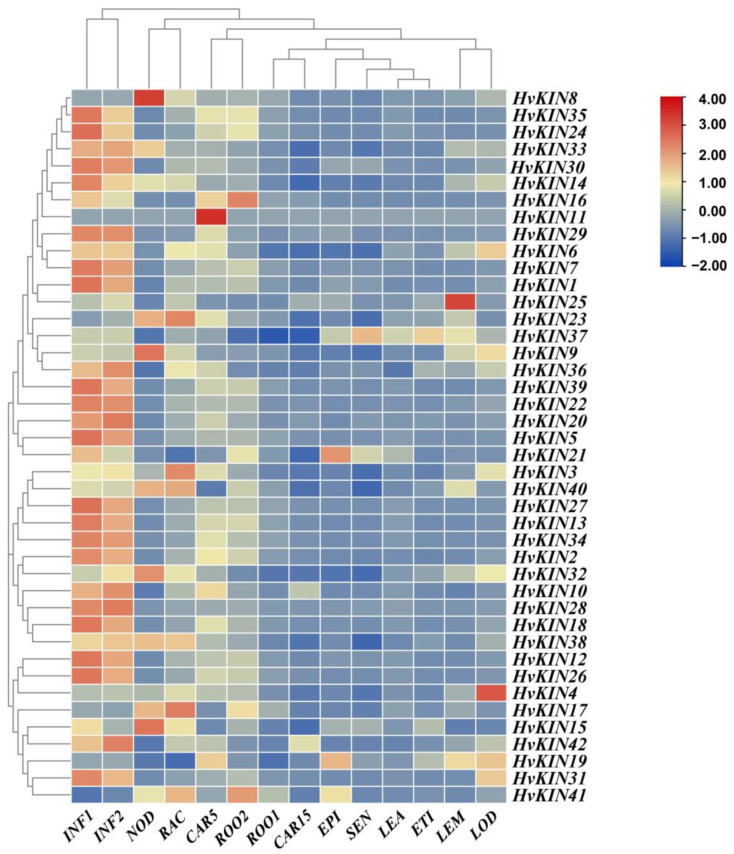
Expression profiles of a subset of *HvKIN*s. A graduated color scale from blue to red is used to denote the transcript levels. Expression data were retrieved from IPK (https://apex.ipk-gatersleben.de/apex/f?p=284:10 accessed on 22 January 2022). The subset of *HvKIN* genes’ hierarchical clustering and normalized expression levels were both adjusted using logarithmic functions. The heatmap was produced using the TBtools software. INF1: Young developing inflorescences (5 mm); INF2: Developing inflorescences (1–1.5 cm); NOD: Developing tillers, 3rd internode (42 DAP); RAC: Inflorescences, rachis (35 DAP); CAR5: Developing grain (5 DAP); ROO2: Roots (28 DAP); ROO1: Roots from seedlings (10 cm shoot stage); CAR15: Developing grain (15 DAP); EPI: Epidermal strips (28 DAP); SEN: Senescing leaves (56 DAP); LEA: Shoots from seedlings (10 cm shoot stage); ETI: Etiolated seedling, dark cond. (10 DAP); LEM: Inflorescences, lemma (42 DAP); LOD: Inflorescences, lodicule (42 DAP).

To preliminarily dissect the functional role of *HvKIN*s in the barley developmental process, a spatio-temporal expression profile of *HvKINs* was constructed with a hierarchical clustering model with the expression data of barley using different periods as well as other tissues (seeds, roots, stem, leaves, flowers, and fruits at different developmental stages) using the IPK (https://www.ipk-gatersleben.de/en/ accessed on 22 January 2022) (Figure 8). As shown in Figure 8, most of the *HvKINs* genes were highly expressed in younger flowers (INF1, INF2), and *HvKINs* were more highly expressed in immature tissues than in older ones. The high expression of kinesin in young flowers is due to the high cell division in young tissues and the role of kinesin in material transport and mitosis [45]. The majority of the *HvKINs* did not show tissue-specific expression; it indicates they played an important role in all growth and developmental stages. However, some members had tissue specific expression patterns; for instance, *HvKIN8* is specifically highly expressed in developing tillers, *HvKIN11* is highly expressed in developing grain, *HvKIN25* is highly expressed in the lemma, and *HvKIN4* is highly expressed in the lodicule.

**Figure 9:**
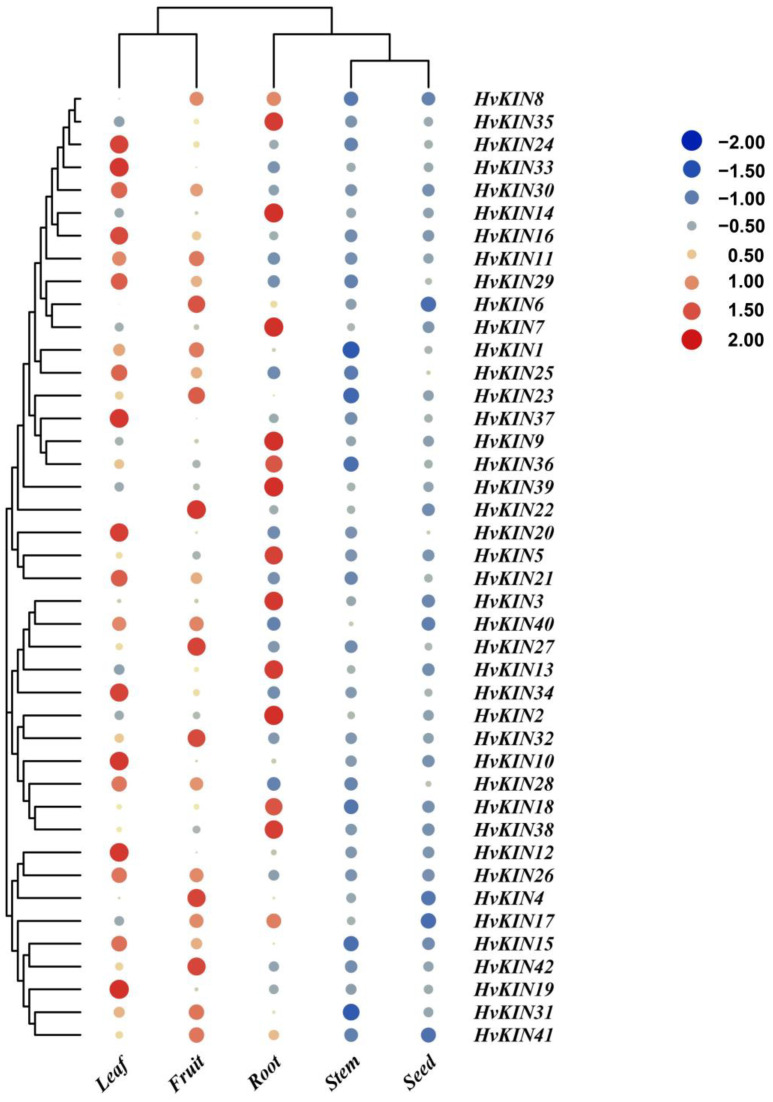
QRT-PCR analysis of the expression level of the *HvKIN*s in different organs of barley. The data summarized at least three biological replicates.

To better understand the potential impact of *HvKIN* genes in different tissues, the expression levels of forty-two genes were determined by QRT-PCR in barley using seedling roots (2 cm shoot stage), stems, leaves, unripe fruit (two-week-old fruiting) and mature seeds (Figure 9). As shown in Figure 9, most of the *HvKIN* is highly expressed in the leaf, roots from seedlings, and inflorescences with lower expression in stem and mature seeds. *HvKIN2*, *HvKIN3*, *HvKIN5*, *HvKIN7*, *HvKIN8*, *HvKIN9*, *HvKIN13*, *HvKIN14*, *HvKIN18*, *HvKIN35*, *HvKIN36. HvKIN38* and *HvKIN39* were highly expressed in roots from seedlings. Kinesins may play an important role in young tissues.

### 3.8. Analysis of HvKINs Expression in Response to ABA and GA3 Treatment

**Figure 10:**
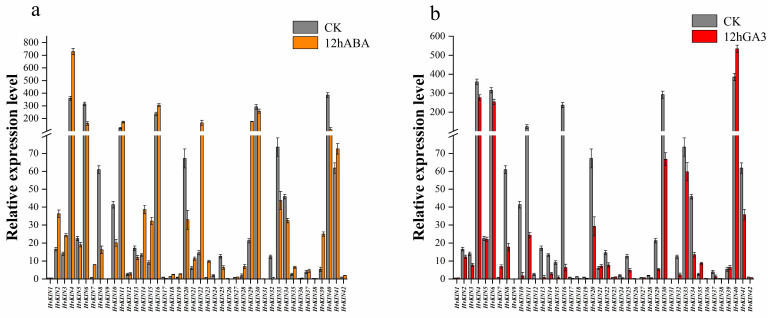
Gene expression analysis of *HvKINs* after ABA and GA3 treatment. The data summarized at least three biological replicates. (**a**) HvKINs gene relative expression in barley after 12 h of ABA treatment. Orange represents the ABA therapy group, while gray represents the control group. (**b**) Relative expression of HvKINs genes in barley following a 12-h GA3 treatment. Red represents the GA3 treatment group and gray served as the control group.

**Figure 11:**
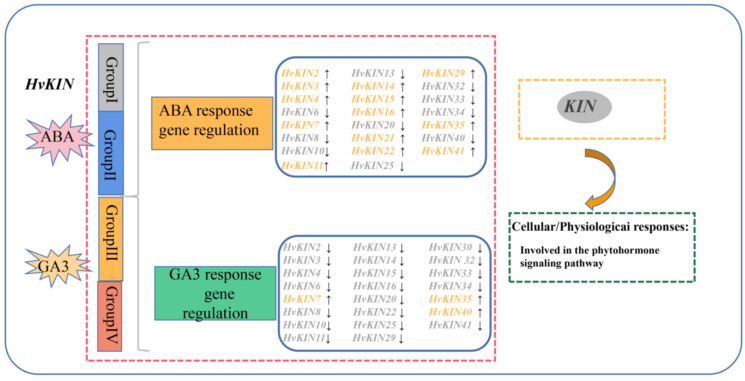
A model for summarizing the role of *HvKINs* based on gene expression profiles in barley.

The cis-element analysis identified several ABA and GA3-responsive elements in the promoters of selected *HvKIN* genes. We analyzed the transcript levels of forty-two kinesin genes after 12 h of treatment using ABA and GA3 sprays on leaves at 30 days of growth, respectively. The results show that half of the kinesins responded to ABA and GA3 treatment (Figure 10, Supplementary Figure S3). Twenty-two of these genes are regulated by both ABA and GA3, but in different ways. After ABA treatment, the expression of 14 *HvKINs* was significantly up-regulated (>2-fold) and 11 *HvKINs* were significantly down-regulated (<2-fold). However, 20 *HvKINs* expression was significantly down-regulated and three *HvKINs (HvKIN7*, *HvKIN35* and *HvKIN40*) expression was significantly up-regulated upon GA3 treatment. Twenty-two *HvKINs* regulated by both ABA and GA3, ten *HvKINs* (*HvKIN 6*, *HvKIN8*, *HvKIN10*, *HvKIN13*, *HvKIN20*, *HvKIN25*, *HvKIN30*, *HvKIN32*, *HvKIN33* and *HvKIN34*) were significantly down-regulated (<2-fold) simultaneously. In addition, *HvKIN7* and *HvKIN35* were significantly up-regulated (>2-fold) at the same time. These results suggest that these genes with significantly altered expression may be involved in regulating plant hormones. Furthermore, according to previous studies, kinesins and ATP have binding sites to microtubules and are jointly involved in the complex life activities of cells (Figure 11) [46].

### 3.9. Subcellular Localization of Selected HvKINs

**Figure 12:**
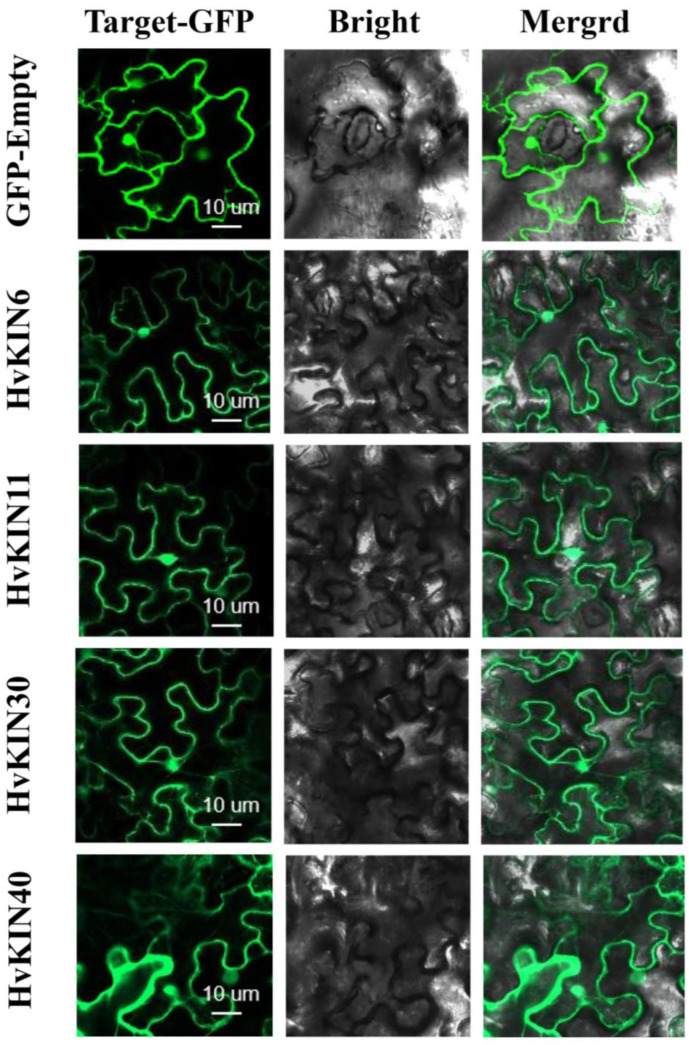
Subcellular localization of selected HvKINs in *Nicotiana benthamiana* (tobacco). Selected HvKIN-GFP fusion protein and using GFP-Empty as the control were independently transiently expressed in *N. benthamiana* leaves and imaged under a confocal microscope. Bars, 10 μm.

The subcellular localization of selected HvKIN proteins (HvKIN6, HvKIN11, HvKIN30 and HvKIN40) were analyzed to assess the possible differences between KvKIN proteins in the barley. We used the heterologous expression of HvKINs fusion proteins in tobacco to analyze their subcellular localization. *HvKIN6*, *HvKIN11*, *HvKIN30*, and *HvKIN40* are mainly localized to the cell membrane and nucleus and may be associated with material transport and cell division in plant cells (Figure 12).

## 4. Discussion

Kinesins are important microtubule-based motor proteins involved in the intracellular transport of substances and also in chromosome division and biological signaling during cell division [47,48,49]. Research on plant kinesins lags behind that of animals and fungi. The reason is not only because plants have evolved a unique kinesin family, but the number of family members is far more than that of animals. The identification and analysis of detailed expression characteristics and functions of kinesin family genes in barley remain elusive. The barley genome has been sequenced so that we can systematically study and analyze the barley kinesin family [50,51]. Through the retrieval, analysis, and organization of the *HvKIN* gene family in barley, a total of forty-two *HvKINs* were identified (Figure 2), and all members contain the KIS (tubulin folding cofactor A (KIESEL)) superfamily structural domain (Figure 4). Since rice has a comparable number of genes overall, the HvKIN family likely had a significant role in plant evolution. Using the maximum likelihood method, a phylogenetic tree was constructed by using 42 members from barley, 48 members from rice, and 61 members from *A. thaliana* (Figure 3). Phylogenetic results showed that 42 *HvKINs* were split into ten main branches, which shows that kinesins are conserved between monocotyledons and dicots (Figure 3). The sequences and molecular weights of *HvKINs* vary considerably, but the structural domains and motif composition are conserved (Figure 4, Supplementary Table S3).

Our results suggest that the majority of HvKINs are scattered near the ends of the chromosomes that are gene-rich (Figure 2), which is similar to previous observations for other gene families in barley [35]. One of the main factors in the complexity of the genome and the rapid expansion and evolution of gene families is gene duplication [52,53]. Gene chromosomal distribution demonstrates that at least seven pairs of *HvKINs* have undergone gene duplication (Figure 6), and most of these gene duplication events correspond to segment duplication. Only one pair of HvKINs (HvKIN28 and HvKIN29) belong to tandem replication (Figure 6).

Studies have shown that in different species, different kinesins have specific functions in plant growth. BR HYPERSENSITIVE 1 belongs to the kinesin 13 subfamily. It plays a signaling role in rice development [54]. Distinctive kinesin-14 motors can associate with midzone microtubules to construct mitotic spindles with two convergent poles in *Arabidopsis* [55]. GmMs1 is a soybean fertility-associated kinesin [56]. MoKin5 and MoKin14 encode the conserved kinesin motor proteins that are essential to form and maintain the spindle and properly nucleate the primary hypha to exhibit canonical functions in *Magnaporthe oryzae* during rice infection [57]. In this study, the expression profiles of barley tissues showed significantly higher expression in immature barley fruits compared to other mature tissues and organs (Figure 8 and Figure 9). There are also reports that several kinesins play an important role in early plant fruit development and play an important role in early fruit development in apples and cucumbers [58,59,60,61]. The role of kinesins in early watermelon fruit development has been investigated. It is significantly characterized by the fact that most kinesins are expressed at high levels in early watermelon fruit [38]. Therefore, based on the functional analysis of kinesins, kinesins may promote cell division during fruit development when cell division is vigorous. During cell division, chromosome duplication in the nucleus and rearrangements require kinesin to provide the impetus, leading to increased cell numbers [62,63]. However, it is not clear if kinesins play a role in which processes in plant fruit development, and future research is needed to focus on this area.

Plant hormones are a cluster of small signal molecules that have been sufficiently reported to play important roles in plant growth and development processes. Early studies found that most gene expressions were related to hormones. GDD1/BC12, a kinesin-like protein, plays an important role in regulating *KO2* gene expression levels in the GA biosynthesis pathway to modulate microtubule rearrangement and cell elongation in rice [64]. Barley kinesin has a regulatory effect on hormones. We predicted the 2000 bp sequence upstream of the promoter of the *HvKINs* gene and found significant differences between individual genes (Figure 5). A large number of hormone-responsive elements were included in all cis-acting elements, with gibberellin (TATC-box) and abscisic acid (ABRE) response elements present in most of the family’s genes (Figure 5). Therefore, we investigate the expression levels of kinesins after treatment with ABA and GA3 in barley (Figure 10). The results showed that most gene expressions were affected in varying degrees (Figure 10). In addition, *HvKIN7* and *HvKIN35* were significantly up-regulated (>2-fold) at the same time, indicating that they play a crucial role in response to phytohormone treatment (Figure 9). Taken together, this provides a further theoretical basis for the relationship between plant hormones and gene expression. Conclusively, our work provides clear clues to further investigation of their detailed roles in barley reproductive development and response to hormones influence.

## 5. Conclusions

Barley kinesin genes have an important role in barley development; the precise roles of *HvKIN* gene family members in barley have not yet been elucidated. Here, our genome-wide analysis and characterization of *HvKIN* genes revealed the physical-chemical properties, chromosome location, phylogeny, gene structure, cis-elements, and expression pattern of these genes. Expression profiling of the *HvKINs* gene was performed to reveal the tissue specificity of the *HvKINs* gene and also to analyze the potential role of the *HvKINs* gene in response to hormonal stimulation. We learned that kinesin expression is high in young plant tissues. Therefore, we speculate that kinesins have an important role in early plant development and flowering. Finally, four *HvKINs* genes (*HvKIN6*, *HvKIN11*, *HvKIN30*, and *HvKIN40*) exhibited high expression and potential functions in plant cells. Currently, there is no detailed genomic-wide analysis of the *HvKINs* gene family in barley. These findings will aid future investigations in the evolutionary origin of *HvKINs* as well as functional studies of candidates of *HvKINs* genes for molecular breeding in barley.

## Data Availability

Not applicable.

## Supplementary Materials

The following supporting information can be downloaded at: https://www.mdpi.com/article/10.3390/genes13122376/s1. Supplementary Table S1: List of vectors sequence primers used in this study; Supplementary Table S2: Subcellular localization primers; Supplementary Table S3: Physicochemical properties of *HvKIN* genes and proteins; Supplementary Table S4: Motif sequences identified by MEME tools in *HvKIN* gene family; Supplementary Table S5: Analysis of *HvKIN* duplicated genes from barley. Supplementary Figure S1: HvKINs structure and conserved motif distribution analysis; Supplementary Figure S2: Cis-acting element structures in promoter regions of barley HvKIN family members; Supplementary Figure S3: Gene expression analysis of HvKINs after ABA and GA3 treatment.
