# Supplementary figures and images for: Genome-Wide Identification and Expression Analysis of Kinesin Family in Barley (Hordeum vulgare)
Source: Genes (Basel). 2022 Dec 16;13(12):2376. doi: 10.3390/genes13122376 (PMC9778244; doi:10.3390/genes13122376)

a

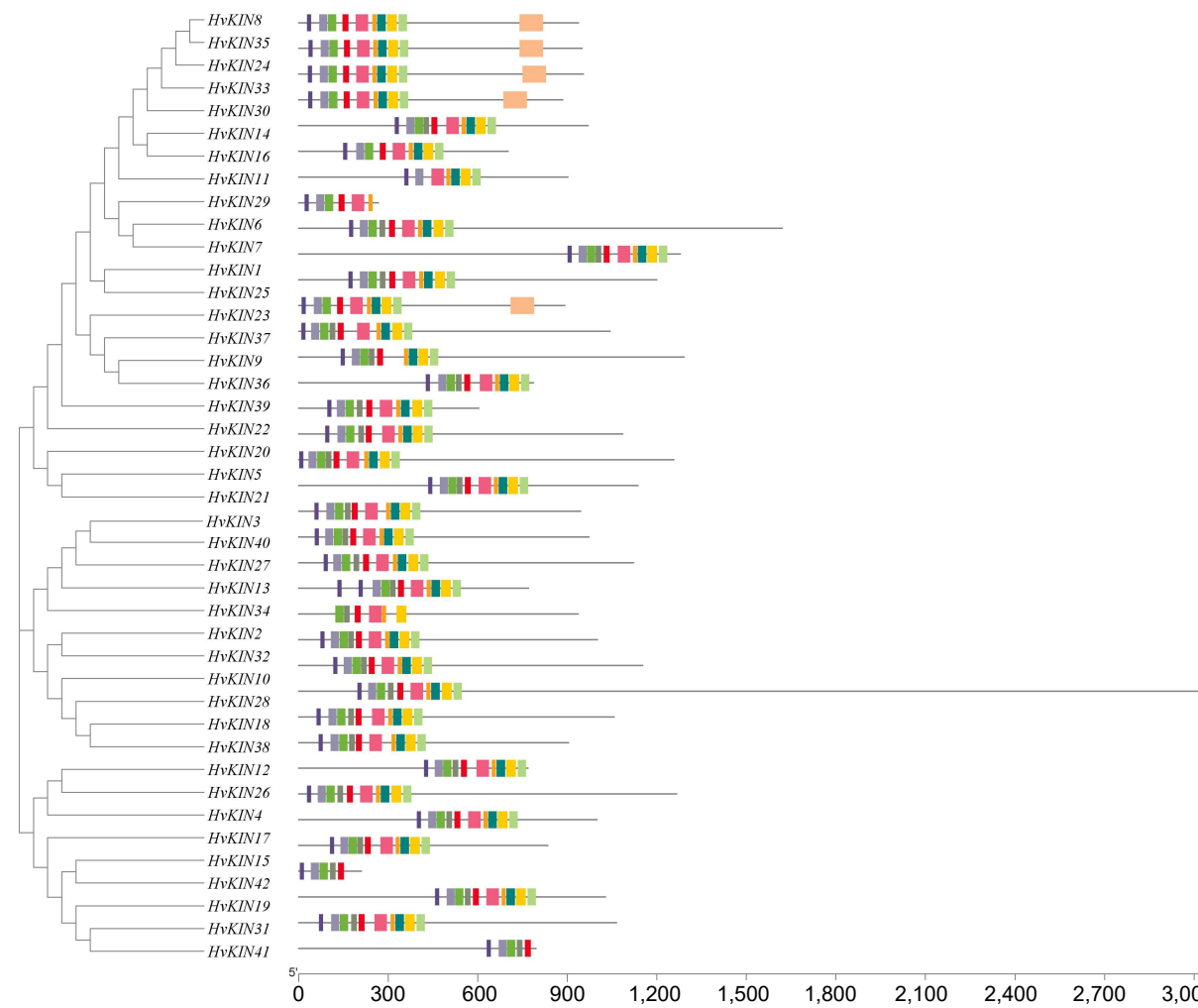

b

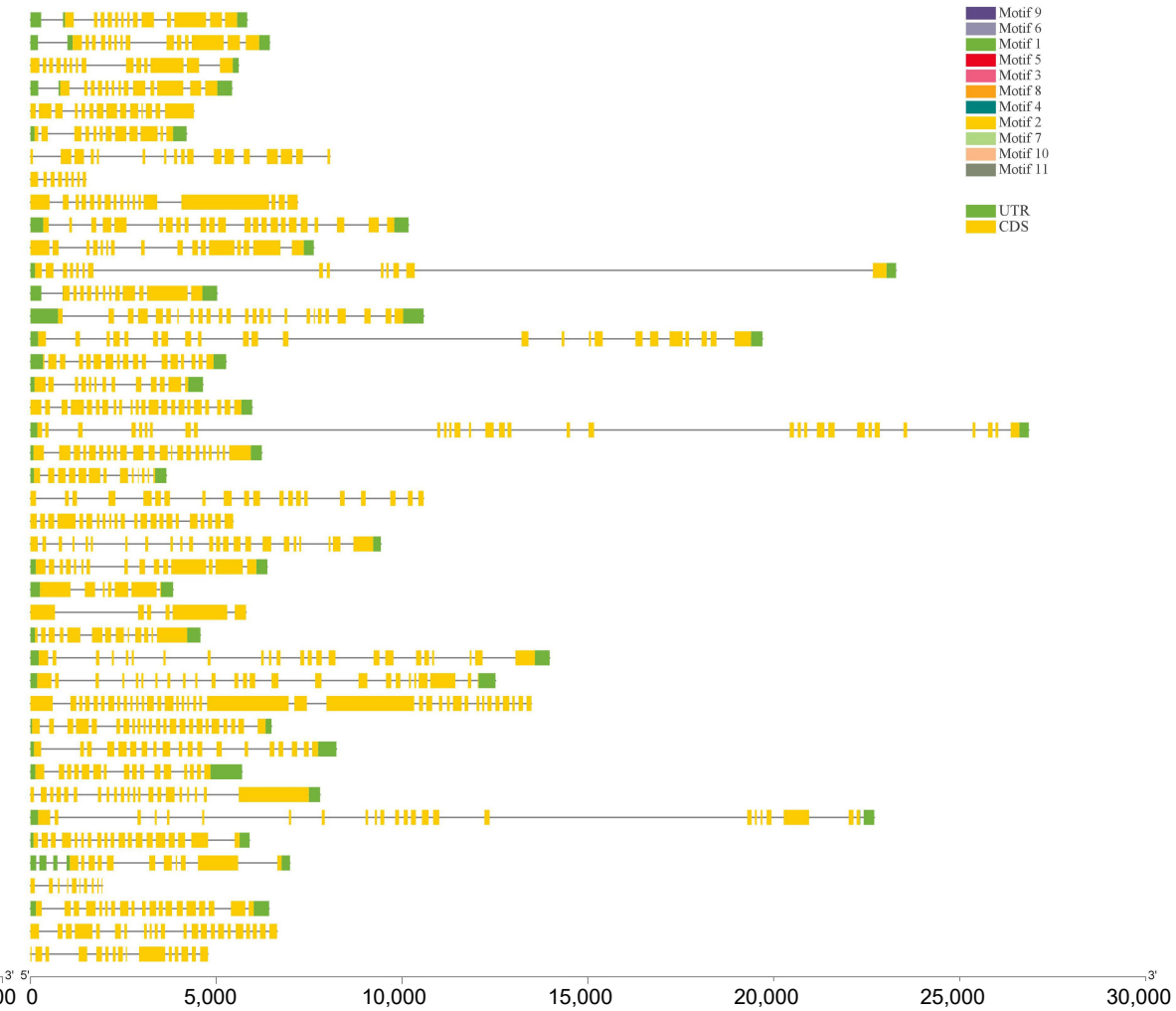

Supplement: Supplementary file 1 [file genes-13-02376-s001.zip › Supplementary Figure S1.pdf]

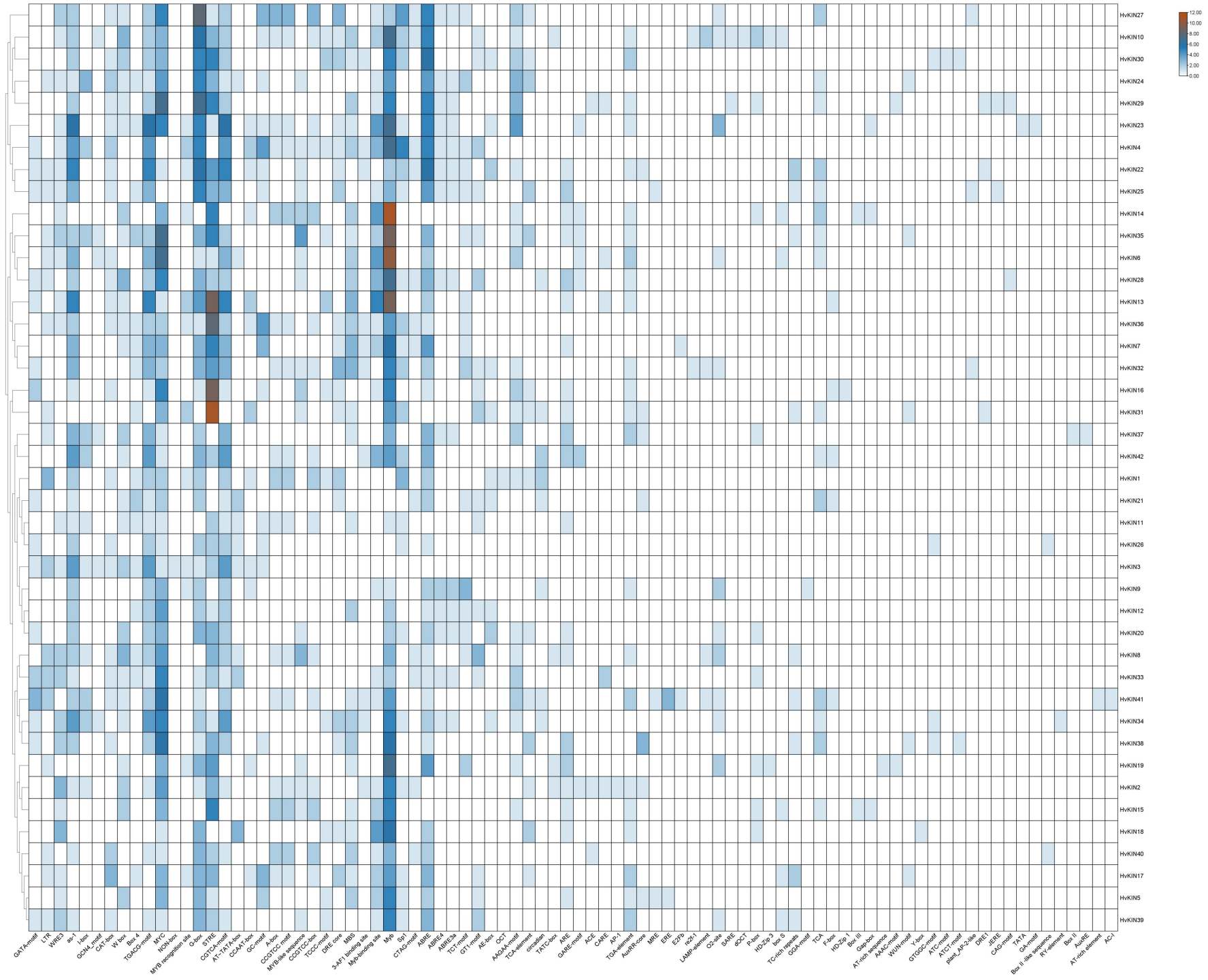

Supplement: Supplementary file 1 [file genes-13-02376-s001.zip › Supplementary Figure S2.pdf]

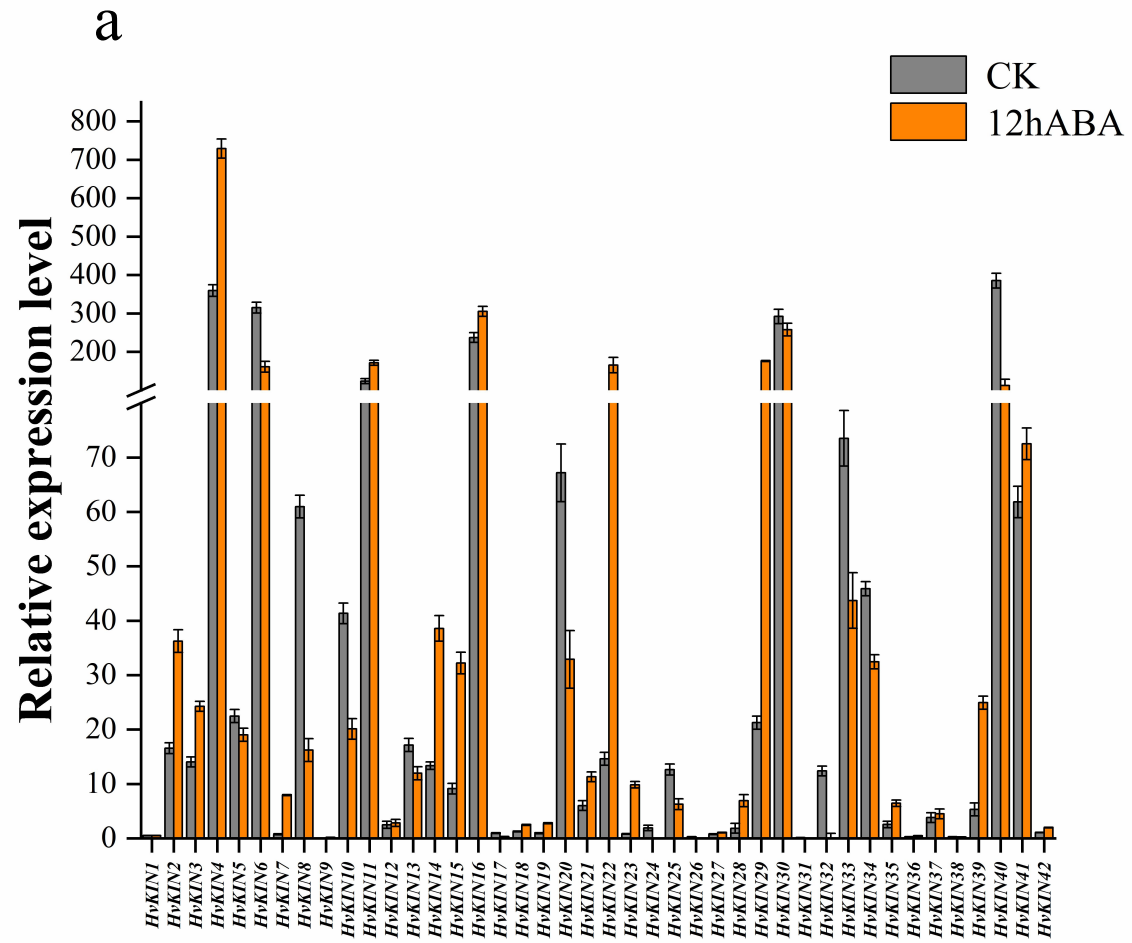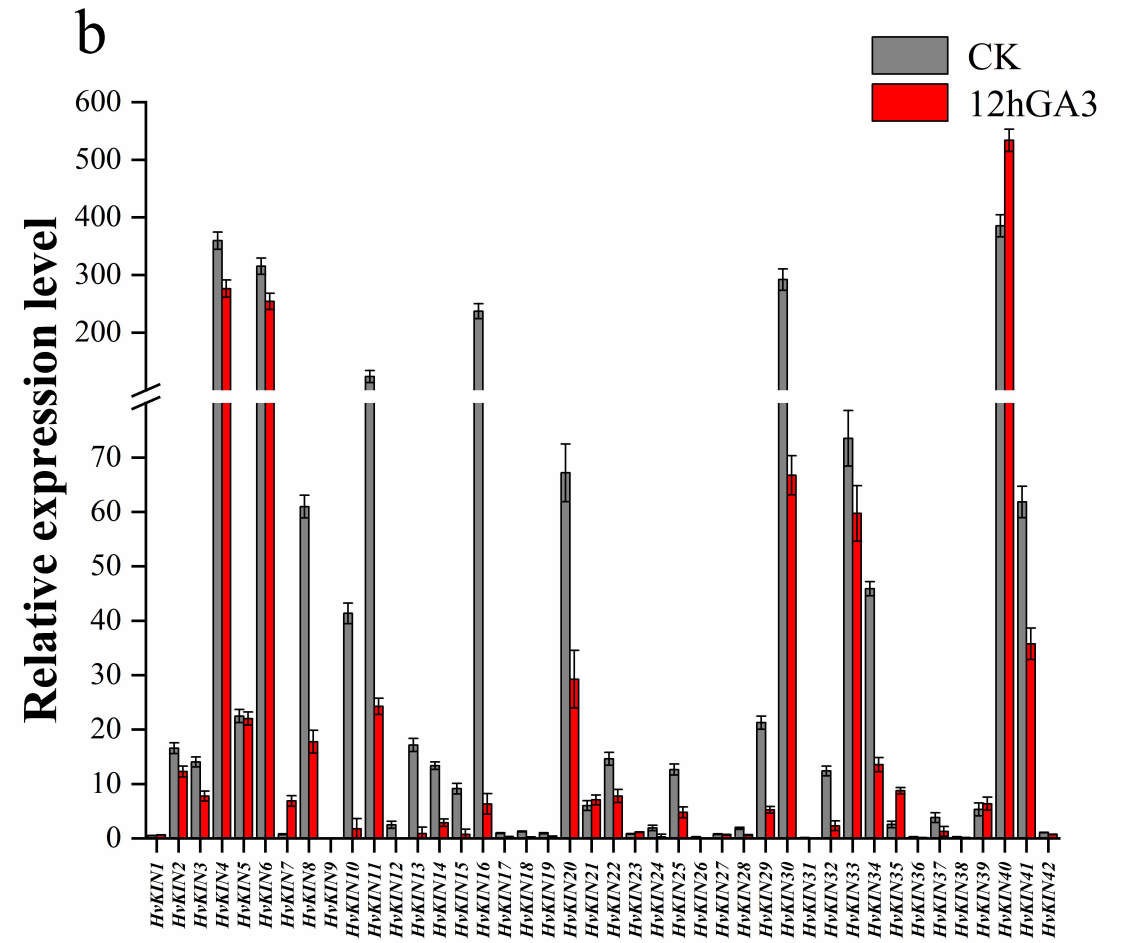

Supplement: Supplementary file 1 [file genes-13-02376-s001.zip › Supplementary Figure S3.pdf]
